# Overcoming Antimicrobial Resistance in Bacterial Keratitis With an Antibiotic-Eluting Contact Lens

**DOI:** 10.1167/iovs.66.15.38

**Published:** 2025-12-12

**Authors:** Liangju Kuang, Amy E. Ross, Lin Chen, Levi N. Kanu, Nikolay Boychev, Alireza Ghaffarieh, Cheng Peng, Paulo J. M. Bispo, Eric G. Romanowski, Daniel S. Kohane, Joseph B. Ciolino

**Affiliations:** 1 Department of Ophthalmology, Schepens Eye Research Institute of Mass Eye and Ear, Harvard Medical School, Boston, Massachusetts, United States; 2 Department of Ophthalmology, Affiliated Hospital of Zunyi Medical University, Zunyi, China; 3 Department of Ophthalmology, Massachusetts Eye and Ear; Infectious Disease Institute, Harvard Medical School, Boston, Massachusetts, United States; 4 The Charles T. Campbell Ophthalmic Microbiology Laboratory, UPMC Vision Institute, Department of Ophthalmology, University of Pittsburgh School of Medicine, Pittsburgh, Pennsylvania, United States; 5 Laboratory for Biomaterials and Drug Delivery, Dept. of Anesthesiology, Boston Children's Hospital, Harvard Medical School, Boston, Massachusetts, United States

**Keywords:** antimicrobial resistance, MRSA keratitis, drug-eluting contact lens, moxifloxacin, ocular drug delivery

## Abstract

**Purpose:**

Bacterial keratitis (BK) is a leading cause of corneal blindness globally, with an alarming rise in resistant infections. Current standard-of-care antibiotic eye drops or ointments suffer from low bioavailability and often fail to achieve therapeutic concentrations in cornea. This study evaluated a therapeutic contact lens (M-TCL) designed to provide sustained, localized high drug concentrations of moxifloxacin—a commonly used broad-spectrum antibiotic—as a potential treatment for drug-resistant BK.

**Methods:**

M-TCLs were engineered by encapsulating a thin moxifloxacin-polymer film within periphery of a contact lens hydrogel. Pharmacokinetics were assessed in rabbit ocular tissues and serum. Efficacy was evaluated using a rabbit keratitis model induced by clinical methicillin-resistant *Staphylococcus aureus* (MRSA) isolates that displayed in vitro resistance to moxifloxacin (minimum inhibitory concentration = 8 µg/mL). Biocompatibility was assessed through Draize testing and histological analysis.

**Results:**

In vitro, M-TCLs retained physical properties comparable to commercial contact lenses, sustained moxifloxacin release over 24 hours, and remained stable in a hydrated state for 12 months, enabling on-demand use. In rabbits, M-TCLs maintained higher corneal drug concentrations at all time points over 24 hours than the peak concentration from moxifloxacin drops (*P* = 0.03), with a 32-fold higher peak drug concentration and at least 11.3-fold greater area under the concentration-time curve (AUC_(0–24 h)_) in cornea. M-TCLs demonstrated bactericidal efficacy against MRSA keratitis with bacterial reduction of 3.86-log CFU/cornea (*P* < 0.0001), and mitigated keratitis-associated inflammation with lower aqueous humor protein levels (*P* = 0.01), compared to hourly moxifloxacin drops. M-TCLs were safe in rabbits.

**Conclusions:**

M-TCLs offer a promising therapy for BK and may serve as a potential therapy for drug-resistant BK.

Bacterial keratitis, a potentially sight-threatening infection of the cornea, presents significant treatment difficulties.^1^^,^^2^ Delayed eradication of the infection can result in severe complications, including corneal scarring, perforations, intraocular spread of the infection, and even permanent blindness or loss of the affected eye.^1^ Compounding these difficulties is the alarming rise in multidrug-resistant ocular infections, particularly resistance to first-line antibiotics such as fluoroquinolones (FQs) among ocular strains of methicillin-resistant *Staphylococcus aureus* (MRSA).^2^^,^^3^ The avascular nature of the cornea and the restrictive blood-aqueous humor barrier limit the efficacy of systemic antibiotics for treating bacterial keratitis. With no alternative ocular drug delivery systems commercially available, treatment currently relies on empiric therapy with antibacterial eye drops or ointments, typically commercial FQs or fortified antibiotics compounded in specialized pharmacies.^4^^,^^5^ However, the effectiveness of eye drops or ointments is hampered by poor bioavailability and patient adherence.^6^^,^^7^

The cornea's unique anatomy—comprising hydrophobic epithelial and endothelial layers alongside a hydrophilic stromal layer—creates significant barriers to drug penetration, restricting the transcorneal transport of both hydrophobic and hydrophilic drugs.^6^ Additionally, a significant portion of the medication applied via eye drops is rapidly eliminated from the ocular surface because of tear dilution, blinking, drainage, and basal or reflex lacrimation.^8^ Despite intensive dosing regimens—as often as every 15 minutes initially and hourly thereafter throughout the day and night,^1^ achieving and maintaining therapeutic drug levels in the cornea remains challenging. The demanding regimens also impose a considerable burden on patients, significantly affecting their quality of life. Unsurprisingly, non-adherence rates are as high as 51%,^9^ further compromising the treatment outcome. Although ophthalmic ointments extend drug retention time in the precorneal area, their thick texture can cause blurred vision and patient discomfort, potentially reducing treatment adherence. Their poor solubility can also hinder the penetration of certain drugs into the cornea, which is essential for effective treatment.^7^

A novel drug delivery method capable of sustaining localized antibacterial drug levels in the cornea presents a promising solution to therapeutic outcomes and combating antibiotic resistance.^10^ Such an approach could achieve drug concentrations exceeding the established susceptibility thresholds against bacteria, especially for FQs, whose antimicrobial efficacy relies on both the drug concentration relative to the minimum inhibitory concentration (MIC) and the duration of exposure.^11^ Thus, even if a bacterium is classified as resistant to a specific antibiotic according to the Clinical Laboratory Standards Institute (CLSI) guidelines, this strategy holds the potential to overwhelm the evolved cellular resistance mechanisms, enabling the eradication of the infection.^12^ Additionally, simplifying the treatment regimen to reduce the frequency of applications could enhance patient adherence, an essential factor in improving therapeutic efficacy. In literature, multiple antibiotic ocular delivery systems have been proposed for bacterial keratitis treatment, including lipid-based nanocarriers,^13^ polymeric nanoparticles,^14^^–^^17^ in situ gels or hydrogels,^18^^-^^23^ ocular inserts/implants,^24^^–^^26^ and drug-eluting contact lenses.^7^^–^^39^ Although many of these methods have improved bioavailability, few have demonstrated sufficient bactericidal effects (≥99.9% bacterial killing) even against susceptible strains, to the best of our knowledge.^23^^,^^39^ Maintaining therapeutic drug levels in target tissues for extended durations remains a critical unmet need, particularly for treating antibiotic-resistant bacterial keratitis.

A contact lens system incorporating an encapsulated drug-polymer film shows promise in addressing these challenges while remaining user-friendly for patient self-application.^8^^,^^40^^–^^44^ Herein, a novel moxifloxacin-eluting therapeutic contact lens (M-TCL) was designed to deliver high concentrations of moxifloxacin directly to the cornea for improved bacterial keratitis treatment. The M-TCL contains a thin, ring-shaped moxifloxacin-polymer film encapsulated within the periphery of a methafilcon hydrogel (Fig. 1A), a widely used material in commercial soft contact lenses.^45^ Moxifloxacin, a fourth-generation FQ antibiotic, was chosen for its broad-spectrum activity, particularly against keratitis-causing pathogens, and its favorable safety profile.^46^ Ethocel ethylcellulose, a biocompatible and physiologically inert polymer,^47^ was selected as the drug delivery matrix for its prolonged stability, attributed to its negligible degradation under physiological and ambient conditions.^48^ The physical properties, in vitro drug release, stability, biocompatibility, and pharmacokinetics of the M-TCL, as well as its in vivo efficacy, were investigated. Pharmacokinetics were evaluated in rabbit ocular tissues and serum, whereas efficacy was tested in a rabbit keratitis model induced by intrastromal injection of FQ-resistant MRSA. Additionally, the safety and biocompatibility of M-TCL were assessed through ocular irritation and histological studies in healthy rabbits.

**Figure 1.**
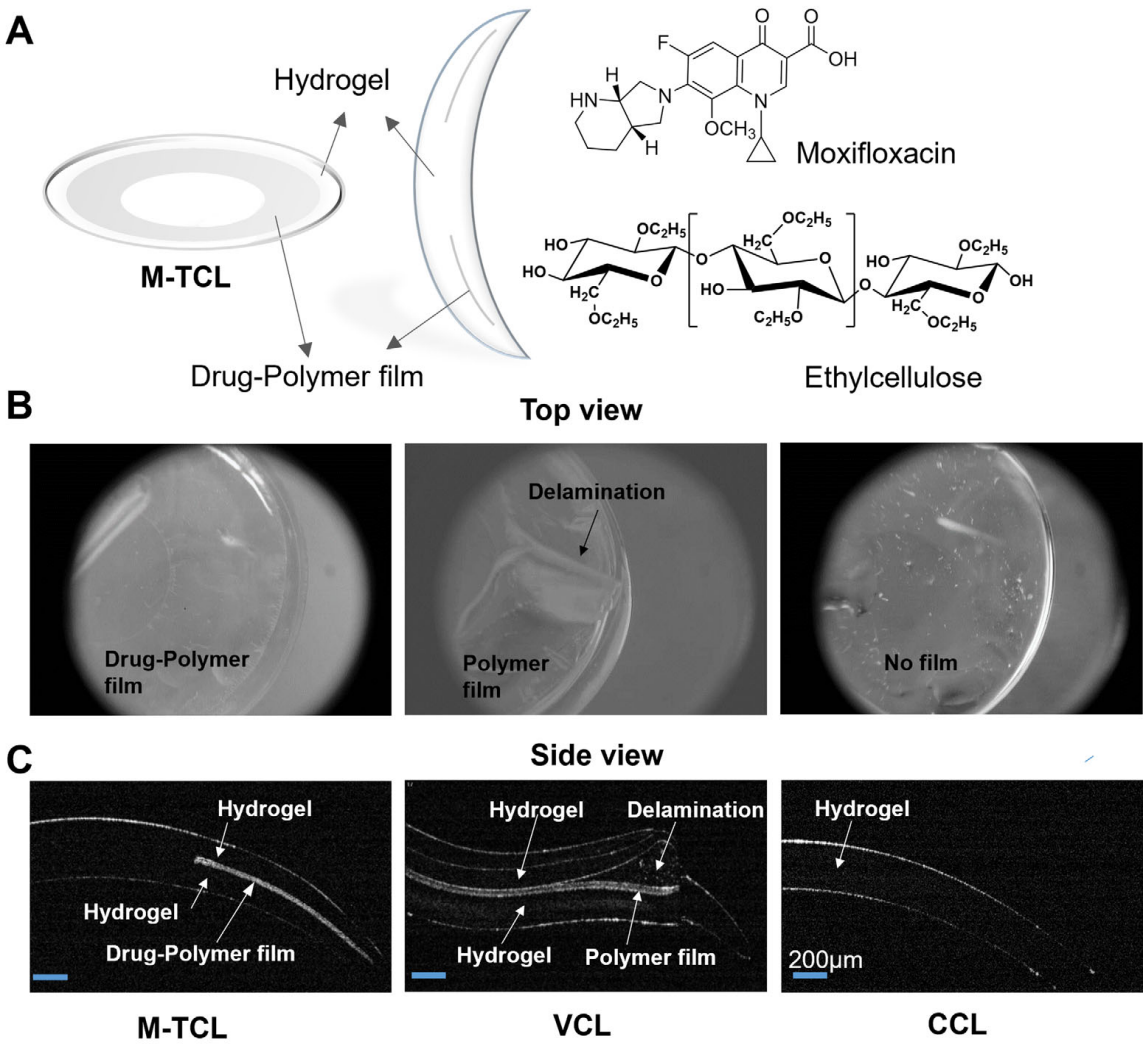
The M-TCL. **(A)** Schematic of M-TCL showing the encapsulated ring-shaped drug (moxifloxacin)-polymer (ethylcellulose) film and central aperture to allow for clear vision. Representative OCT **(B)** photos (*top view*) and **(C)** images (*side view*) of the M-TCL, ethylcellulose-film only encapsulated VCL, and commercially available Kontur contact lens (CCL) that have been prehydrated in a moxifloxacin solution with a concentration of 5.8 mg/mL and a pH of 7.4 for 24 hours. VCL is associated with delamination and misshapenness. *Scale bar*: 200 µm. *n* = 4 per group.

## Material and Methods

### Materials

Moxifloxacin free base with purity ≥ 98% was purchased from LKT Labs (St Paul, MN, USA). Moxifloxacin eye drops (0.5%, Vigamox) were obtained from Mass Eye and Ear Pharmacy. Ethocel 4 ethylcellulose (Viscosity: 3.0–5.5 mPa.s, ethoxyl Content: 48–49.5 wt%)^49^ was provided by the Dow Chemical Company (Midland, MI, USA). Chloroform was purchased from EMD Millipore (Darmstadt, Germany), and dibutyl sebacate was purchased from Sigma-Aldrich Corp. (St. Louis, MO, USA). Dry concavity-lathed contact lens blanks, liquid methafilcon, and commercial methafilcon contact lenses were purchased from Kontur Kontact Lens (Hercules, CA, USA). Liquid methafilcon was composed of 97.45%–97.53% (v/v) (2-hydroxyethyl methacrylate) (HEMA), 1.72%–1.80% (v/v) methacrylate acid, and 0.75% (v/v) ethylene glycol dimethacrylate (cross-linker). PBS (pH 7.4) was obtained from Invitrogen (Carlsbad, CA, USA). Tryptic soy broth was purchased from Ward's Science. TNF-α and IL-8 ELISA kits were purchased from Antibodies-online (Philadelphia, PA, USA) and Thermo Fisher Scientific (Waltham, MA, USA), respectively.

### Fabrication of M-TCLs

**Figure 2.**
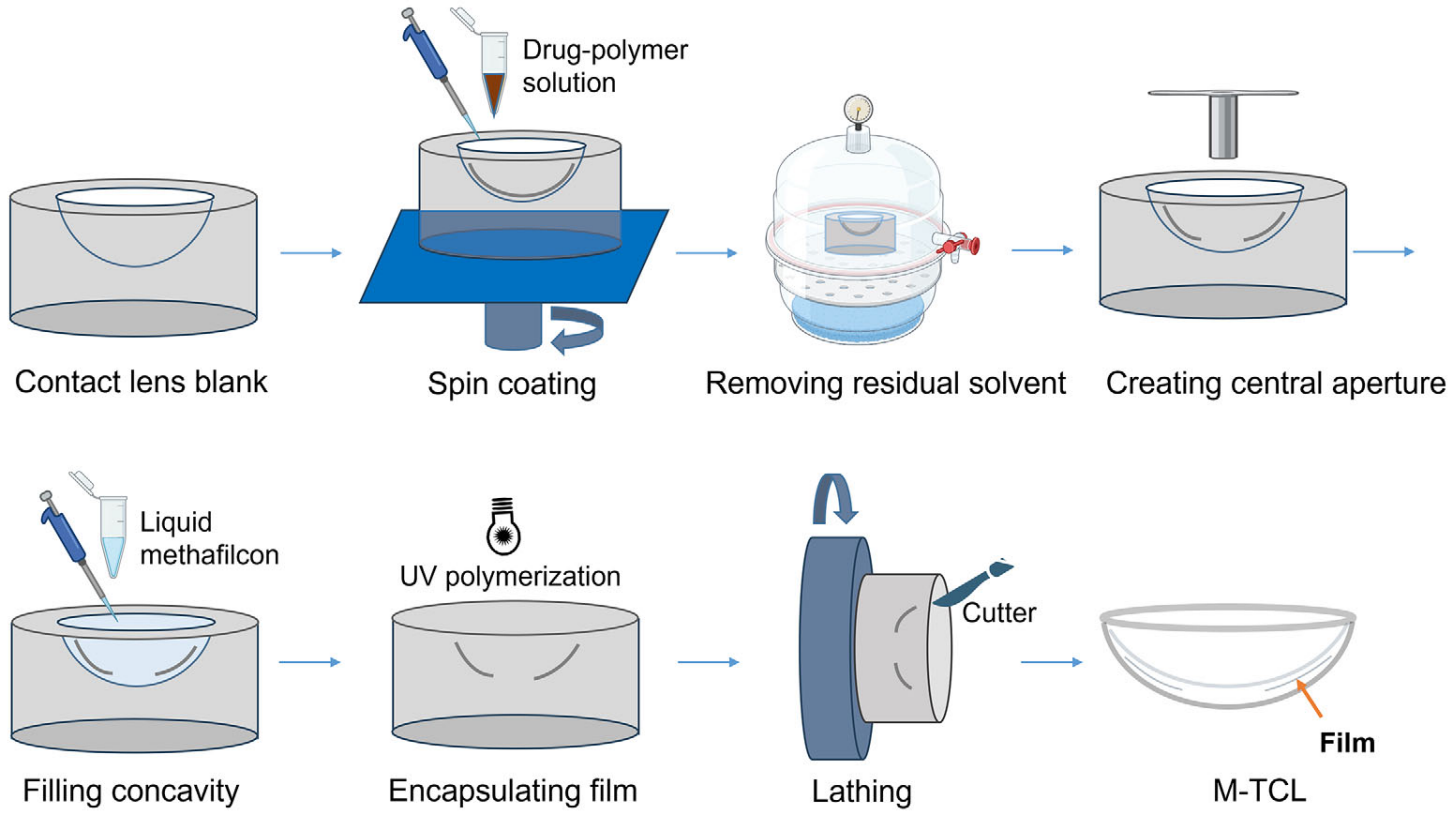
Schematic diagram of the M-TCL fabrication.

Moxifloxacin (21.0% [w/w]), ethylcellulose (63.2% [w/w]), and dibutyl sebacate (15.8% [w/w]) were dissolved in chloroform to form a drug-polymer solution. A 60 µL aliquot of this solution was pipetted into a concavity of a methafilcon lens blank, which had been lathed from a methafilcon cylinder (Fig. 2). The blank containing the drug-polymer solution was then rotated on a spin coater (Best Tools, LLC, St. Louis, MO, USA) at 100 rpm for eight minutes to evaporate the chloroform solvent, leaving behind a uniform drug–polymer film along the concavity walls. A central aperture was created in the film using a four mm derm biopsy punch (Sklar Instruments, West Chester, PA, USA). The contact lens blanks containing the drug-polymer film were placed in a desiccator for seven days to remove residual chloroform. Subsequently, 350 µL of liquid methafilcon formulation was pipetted to fully fill the concavity, totally covering the drug-polymer film. The filled blank was immediately placed in a Zeta 7401 ultraviolet (UV) chamber (Loctite, Rocky Hill, CT, USA) to polymerize and cure the liquid methafilcon. The resulting solid methafilcon cylinder was then lathed into a contact lens, with the drug-polymer film encapsulated within the periphery region of the methafilcon hydrogel.

The lathed CLs were sterilized using a Gamma Cell 220E Cobalt 60 Irradiation Unit (Atomic Energy of Canada Ltd., Ottawa, Canada) with a total dose administration of 25 kGy. Under sterile conditions and protected from light, the lenses were then immersed in one mL moxifloxacin buffered solution (5.8 mg/mL, pH 7.4) for 24 hours to produce the hydrated moxifloxacin-therapeutic CL (M-TCL) for the study. Vehicle contact lenses (VCLs), which contained the ethylcellulose polymer film without any drug, were prepared in a similar manner.

### Optical Coherence Topography

An anterior segment optical coherence topography (OCT), RTVue OCT (Optovue, Fremont, CA, USA), was used to image the lenses and assess their morphology in vitro. Each lens was positioned with the convex side of the lens facing the OCT camera, and Raster scanning imaging was selected to obtain cross-sectional images of the contact lens and the drug-polymer film. Another anterior segment OCT, a spectral-domain OCT (Spectralis, Heidelberg Engineering, Heidelberg, Germany), was used in vivo to evaluate M-TCLs worn by rabbits under general anesthesia.

### Water Content

Lenses were incubated at 37°C in PBS for 48 hours. After incubation, the lenses were then removed from PBS, with excess PBS blotted by Kimwipes, and their weights were measured (W_wet_). Subsequently, the lenses were lyophilized for three days and weighed again (W_dry_). The water content of the lenses was expressed as (1 − W_dry_/W_wet_) × 100%.^50^

### Light Transmission

Commercial contact lenses and M-TCLs were removed from the storage solution and rinsed with PBS. Light transmission through each hydrated lens was then evaluated using a SpectraMax M3 microplate reader.^43^ The light transmission of each lens was calculated as the mean transmittance across the visible light spectrum (450–700 nm).^51^ The overall average transmission was then determined by averaging the values from four individual lenses per group.

### Mechanical Properties

The tensile tests of the contact lenses were performed at room temperature using a Mark-10 ESM 303 motorized test stand (Mark-10 Corporation, Copiague, NY, USA). Before testing, samples were removed from the storage solution and rinsed with PBS. Strip-shaped specimens, with a width of ∼5 mm and a total length of ∼9 mm, were stretched at a constant rate of 0.5 mm/s. The Young's modulus was calculated from the linear derivative of the stress–strain curve in the low stiffness range (<10% strain).^43^^,^^52^ Four independent measurements were conducted for each group.

### In Vitro Release and Storage Stability Study

To carry out the in vitro release study, individual lens (*n* = 4) was removed from the moxifloxacin solutions, immersed in five mL PBS, and then placed in an incubator shaker at 37°C and 64 rpm. At predetermined times, the lenses were removed and placed in five mL of fresh PBS. Aliquots of the PBS release media were sampled and stored at 4°C until drug concentration was quantified by a Dionex ICS 5000 ^+^ HPLC (Thermo Fisher Scientific, Waltham, MA, USA). The gradient mobile phase started at 20% (v/v) acetonitrile and 80% (v/v) of 0.1% (v/v) trifluoroacetic acid and ended at 50% (v/v) acetonitrile and 50% (v/v) of 0.1% trifluoroacetic acid over 15 minutes, back to 20% (v/v) acetonitrile for one minute and equilibrated for one minute. Samples were analyzed on a C18 column (4.6 mm × 25 cm, 5 µm, Harmony) and detected by UV detector at 288 nm.

To investigate the long-term stability of M-TCL in a hydrated state, the lenses (*n* = 4-5 per group) were individually stored in the moxifloxacin buffered solution (5.8 mg/mL, pH = 7.4) at room temperature in a 16-mm glass tube with light protection for one month and 12 months. Post-storage drug release kinetics were then investigated. To compare the similarity/difference, the similarity factor, f_2_, was calculated.^53^

### Animals

New Zealand white rabbits (both male and female, 3–12 months old, 2.5–5 kg) from Charles River Laboratories (Boston, MA, USA) were used in all animal studies. Female and male rabbits were randomly assigned to experimental groups in approximately equal numbers (unless otherwise specified) before treatment and analysis. General anesthesia was administered by intramuscular injections of 30 mg/kg ketamine, 5 mg/kg xylazine, and 1 mg/kg acepromazine. Buprenorphine hydrochloride (0.05 mg/kg, subcutaneous) was used for pain control. Euthanasia was performed by an intravenous injection of 120 mg/kg Euthasol euthanasia solution. All animal studies were approved by the Schepens Eye Research Institute Institutional Animal Care and Use Committee (protocol number: 2020N000193 for ocular pharmacokinetics; 2020N000192 for in vivo efficacy; and 2021N000159 for in vivo ocular irritation and biocompatibility). All animals were treated according to the Association for Research in Vision and Ophthalmology (ARVO) Statement for the Use of Animals in Ophthalmic and Vision Research (ARVO Handbook, 1993). Tarsorrhaphies were used to improve contact lens retention. Similar to previous reports,^8^^,^^40^^–^^42^^,^^44^ tarsorrhaphies were performed on the right eye in all study groups, irrespective of assignment to contact lens treatment, to avoid any confounding that could result from the tarsorrhaphy. The contralateral left eye remained untreated and served as a healthy control.

### Ocular Pharmacokinetics

In the pharmacokinetic study, all rabbits underwent a permanent lateral tarsorrhaphy on the right eye. Under general anesthesia, the lateral third of the eyelid was approximated using 6-0 Vicryl sutures. The rabbits either wore the M-TCL or received intensive moxifloxacin eye drops (one drop every 15 minutes for one hour). At predetermined time points (*n* = 6 per time point), aqueous humor (100 µL) was removed under general anesthesia by placing a 30-gauge needle in the superior cornea in a manner that created a self-healing wound. Rabbits wore a different M-TCL or were redosed with moxifloxacin drops at each time point to allow for aqueous humor replenishment, as well as for practical and humane reasons. A minimum three-day washout period was used between time points to prevent confounding results.

To evaluate the drug concentrations in other ocular tissues (*n* = 4), the rabbits were euthanized at predetermined time points (two, four, eight, 18, and 24 hours for M-TCL; 0.5 hour, two and four hours for moxifloxacin drops). Eyes were then enucleated, frozen at −144°C, and dissected into cornea, iris, lens, retina, choroid, sclera, and vitreous humor. The drug concentrations in tissues were quantified by HPLC with fluorescence detection (method detection limit: 1.5 ng/mL). The measured moxifloxacin concentration was further plotted against time. The maximum concentration (C_max_) and time to reach C_max_ (t_max,_) after dosing were recorded as observed. The area under the concentration time curve for 24 hours (AUC_(0–24 h)_) for M-TCL was calculated using linear trapezoid rules.^54^

$$\begin{array}{c} AUC=\sum_{i=0}^{N}\frac{C_{i}+C_{i+1}}{2}\;\left(t_{i+1}-t_{i}\right) \end{array}$$

*C_i_* and *C_i_*_+1_ are the corresponding sample concentrations. *t_i_*_+1_ and *t_i_* are the times of sampling. Assuming continuous administration of moxifloxacin drops every 15 minutes for 24 hours (total 96 drops), the AUC_(0–24 h)_ for moxifloxacin drops was estimated to be 24 times the C_max_.

### Serum Pharmacokinetics

One mL of blood was drawn from anesthetized rabbits approximately 30 minutes after administration of the fourth moxifloxacin drop (every 15 minutes for one hour) and at two, four, eight, 18, 24, and 72 hours after the M-TCL placement during the ocular pharmacokinetic study and in vivo biocompatibility study. The collected blood was spun in a centrifuge at 4°C at 4000 rpm for 10 minutes, and the separated serum was collected and stored at −144°C. Moxifloxacin concentrations in the serum were measured by HPLC with fluorescence detection.

### Clinical Keratitis Isolate and Inoculum Preparation

A FQ-resistant MRSA (MRSA2757) was recovered from a patient presenting with infectious keratitis and stored at −80°C in the biorepository using tryptic soy broth plus 20% glycerol. The human tissue experiments complied with the guidelines of the ARVO Best Practices for Using Human Eye Tissue in Research (Nov 2021). Protocols for obtaining discarded isolates with waived informed consent were approved by the Mass General Brigham Institutional Review Board (Protocols 214 2021P000695, approved 5/18/2021, and 2019P001001, approved 04/11/2019). The MIC of moxifloxacin against the MRSA clinical isolate was determined as the lowest concentration at which no turbidity was observed, using reference broth microdilution as per the CLSI standard.^55^ Frozen stocks of the MRSA isolate were grown on 5% sheep blood agar plates at 37°C. After 18 hours, five colonies were suspended in tryptic soy broth at 120 rpm and 37°C to the turbidity of a 0.5 McFarland Standard. Turbidity was measured with a SpectraMax spectrophotometer (Molecular Devices, San Jose, CA) and correlated to CFU counts. Concurrently, a standard colony count was measured on the turbid bacterial suspension. Cultures were diluted and loaded into gastight 100 µL gastight syringes (Hamilton Company, Reno, NV, USA) for infection inducement in the in vivo efficacy study.

### Efficacy in a Rabbit Model of MRSA Keratitis

To induce MRSA keratitis, the right eye of anesthetized rabbits was injected intrastromally with the 25 µL MRSA2757 isolate (2000 total CFUs) using a 31-gauge, 1/2-inch needle attached to a 100-µL Hamilton syringe. After this injection, all rabbits (right eye) subsequently received a central temporary tarsorrhaphy covering about 40% of the eye using 5-0 Nylon sutures. After a four-hour incubation as per the established model,^3^^,^^56^^,^^57^ the rabbits were randomly assigned to a baseline infection group and five treatment groups. Animals in the baseline infection group were examined using slit lamp biomicroscopy with a Topcon DC-3 digital camera attachment (Topcon Optical Company, Tokyo, Japan) and then immediately euthanized to determine baseline bacterial counts (*n* = 7). The five treatment groups were as follows: (1) no treatment (*n* = 8); (2) hourly moxifloxacin (0.5%) eye drops (*n* = 7); (3) the new M-TCL (*n* = 8); (4) M-TCL that has been pre-worn by a healthy rabbit for eight hours before being placed on infected eyes (M-TCL_pre-worn8h_, *n* = 4); and (5) M-TCL that has been pre-worn by a healthy rabbit for 18 h ( M-TCL_preworn18h_, *n* = 4). The treatment duration was set at four hours according to previous studies,^3^^,^^56^^,^^57^ as well as for practical and humane reasons. After the treatment, all rabbits underwent a follow-up slit lamp examination and were then euthanized to harvest the aqueous humor and the cornea.

For CFU measurement, an 8.5 mm corneal button was excised from each infected eye and homogenized in one mL PBS using an Omni Bead Ruptor (Omni International, Kennesaw, GA, USA). After homogenization, the supernatants were collected by centrifugation at 1500*g* for five minutes, and then aliquots of supernatants were cultured after serial dilutions on tryptic soy agar plates in duplicate at 37°C. After 18 hours of incubation, the CFUs of the homogenized corneas were quantified by two masked examiners.

For drug concentration measurements, the residual peripheral cornea—remaining after excising an 8.5 mm central corneal button—was collected and stored at −144°C for subsequent drug quantification. Moxifloxacin concentrations were then measured using HPLC, following the protocol described in the Ocular Pharmacokinetics section above. The concentrations of cytokines (i.e., TNF-α and IL-8), in the undiluted supernatants were measured using commercial ELISA kits.^58^^,^^59^ The total protein concentrations in the aqueous humor were measured using a Bradford Assay (ThermoFisher Scientific, Waltham, MA, USA). Contralateral eye samples were randomly selected from each treatment group and analyzed as a single group to serve as negative controls.

To objectively and quantifiably evaluate the clinical signs, the obtained slit lamp photographs were randomized and independently graded by two masked corneal specialists following an established method.^60^ Three parameters were assessed to determine the severity of the infection: conjunctival injection, conjunctival edema (chemosis), and corneal infiltration. Each parameter was assigned a grade from zero (normal) to four (maximally severe) by each observer. The final score for each parameter for each eye was calculated by averaging the scores given by the two observers. The three grades were then added together to get a total score for each eye.^60^

### In Vivo Ocular Irritation Study

The potential adverse effects of M-TCLs were accessed using the Draize test in rabbits.^61^ M-TCLs were immersed in roughly 0.85 mL of either sterile saline solution or cottonseed oil (Spectrum Chemical, New Brunswick, NJ, USA) at 37°C for 72 hours to capture polar and non-polar leachables, respectively. The volume of saline solution or cottonseed oil was chosen to provide a surface-to-volume ratio of six cm^2^/mL.^61^ A 100 µL aliquot of extract was instilled in the lower conjunctival cul-de-sac of the right (test) eye in three rabbits (one male and two female healthy rabbits). The eyelids were closed manually for 30 seconds. The contralateral left eye remained untreated and served as a control. Both eyes were physically examined by an ophthalmologist using slit lamp biomicroscopy (a Topcon DC-3 system). Ocular irritation was subjectively graded using the Organization for Economic and Cooperative Development (OECD) scale at pre-defined intervals (one, 24, and 72 hours), after extract instillation [36]. The 0-4 scale evaluated the cornea, iris, conjunctiva, and eyelids, where 0 = normal and higher grades indicated complications, for signs of opacity, ulceration, iritis, hemorrhage, redness/hyperemia, swelling/chemosis, and ocular discharge. Each rabbit had a saline solution extract applied from one lens and cottonseed oil extract applied from one lens after one week.

### In Vivo Biocompatibility

The M-TCL was placed on rabbit's right eye (one male and two female healthy rabbits) for a consecutive 72 hours. A temporary tarsorrhaphy was used to improve M-TCL retention. The contralateral left eye was untreated and served as a control. The rabbits were evaluated daily for signs of discomfort and re-evaluated by a slit lamp biomicroscope. After 72 hours of lens wear, the rabbits were euthanized. The eyes were then removed, fixed in 10% formalin, and embedded in paraffin. Histology slides were stained with hematoxylin and eosin (H&E) to visualize ocular structures.

### Statistical Analysis

Data are expressed as mean ± standard deviation (SD) unless noted and obtained in at least three replicates. The clinical scores are presented as median ± standard deviation. The corneal CFUs from each rabbit in the efficacy studies were converted to log values (i.e., 10^6^ CFU was converted to 6) using a log10 (CFU + 1) transformation, a standard practice in microbiological studies.^3^ Statistical analysis was done by Mann-Whitney U test for continuous data where the results did not follow a normal distribution (namely, the ocular tissue and serum concentrations in pharmacokinetic studies) or Kruskal-Wallis ANOVA for non-continuous data (namely, clinical sores). All normal distribution data were analyzed by Student's *t*-test (the bacterial load and inflammation level comparison between the baseline infection group and each treatment group as described below) or ANOVA followed by Tukey post-hoc tests (the comparison among treatment groups as described below). The significance level for rejection of the null hypothesis was set at 0.05.

## Results

### Development and Characterization of M-TCLs

M-TCLs were designed with a thin, ring-shaped moxifloxacin-polymer film fully encapsulated within the periphery of a methafilcon contact lens (Fig. 1A). The clear central aperture enabled vision unimpeded by the drug-polymer film. The lens fabrication process is described in the section of fabrication of M-TCLs. Briefly, a drug-polymer film was formed on the concave surface of a hydrogel blank via spin coating, and then encapsulated within a hydrogel through UV polymerization, followed by lathing into the contact lens (Fig. 2). Unlike our previously reported drug-eluting contact lenses, which utilized a hydrolyzable polymer matrix (i.e., poly [lactic-co-glycolic] acid; PLGA) and required dry storage,^8^^,^^40^^–^^44^ the M-TCL used Ethocel ethylcellulose within the drug-polymer film. This inert polymer remains stable under physiological conditions,^62^ minimizing drug release because of matrix degradation—even in the aqueous environment required for hydrated contact lenses.

The dry M-TCLs were submerged, hydrated, and stored in a high-concentration buffered moxifloxacin solution (5.8 mg/mL). This approach minimized drug diffusion from the lenses during the hydration and wet storage while enabling additional moxifloxacin absorption from the storage solution. Consequently, the hydrated M-TCLs contained both moxifloxacin incorporated in the drug-polymer film and additional antibiotic absorbed from the hydrating solution. After hydration, the M-TCL exhibited a total diameter of 14.7 mm, with the drug-polymer film encapsulated between an inner diameter of 5.2 mm and an outer diameter of 12.0 mm.

The morphology of the hydrated M-TCL was evaluated using OCT, a high-resolution imaging technique that provides cross-sectional views of soft materials.^63^ Results were compared to those of the VCL, which contains an ethylcellulose polymer film without moxifloxacin, and to a commercially available methafilcon contact lens (CCL), widely used as a bandage lens to protect the cornea from mechanical trauma or disease and to promote healing.^8^ All lenses were hydrated following the protocol outlined above. After hydration, VCLs exhibited misshapenness, and OCT imaging revealed delamination between the polymer film and the methafilcon hydrogel (Figs. 1B, 1C). Because of these structural instabilities, VCLs were deemed unsuitable for in vivo use and were therefore excluded from subsequent studies. In contrast, M-TCLs showed no delamination or misshapenness, which may be attributed to electrostatic interactions between the cationic group in moxifloxacin and the anionic methacrylic acid groups in the methafilcon hydrogel at pH 7.4.^64^ M-TCLs retained the expected contact lens shape and dimensions, comparable to those of CCLs. (Figs. 1B, 1C). The drug-polymer film in M-TCLs, with a thickness of 43 ± 4 µm, formed a uniform, ring-shaped drug delivery zone fully encapsulated within the methafilcon hydrogel (Figs. 1B, 1C).

The water content (percentage swelling) of poly (2-hydroxyethyl methacrylate)–based soft contact lenses, such as methafilcon, is strongly associated with oxygen permeability and lens comfort.^45^ The water content of M-TCLs was measured using a gravimetric method. M-TCLs maintained equilibrium water contents of 54.4% ± 6.4%, comparable to commercial methafilcon contact lenses (54.5% ± 0.4%; *P* = 0.98, *n* = 4, Student's *t*-test).

Light transmittance was measured using a spectrometer.^51^ M-TCLs maintained an average light transmittance through the central aperture (hereafter referred to as central light transmittance) of 94.2% ± 0.3% across the visible spectrum. No significant difference was observed in central light transmittance between M-TCLs and CCLs (94.7% ± 3.0%; *P* = 0.73, *n* = 4, Student's *t*-test). The drug–polymer film zone of M-TCLs showed a significantly lower transmittance compared with CCLs (*p* = 0.0001, Student's *t*-test) but remained semitransparent, with 82.4% ± 2.0% transmittance across the visible spectrum.

Tensile testing was performed to determine the elastic modulus, a key parameter reflecting resistance to deformation and influencing lens fit and handling characteristics.^52^ M-TCLs had an elastic modulus of 0.50 ± 0.05 MPa, comparable to that of CCLs (0.45 ± 0.04 MPa; *P* = 0.15, *n* = 4, Student's *t*-test). This similarity was reflected in the practically indistinguishable handling observed when placing and removing both M-TCLs and CCLs in rabbits, supporting their suitability for clinical use.

### In Vitro Release and Storage Stability

**Figure 3.**
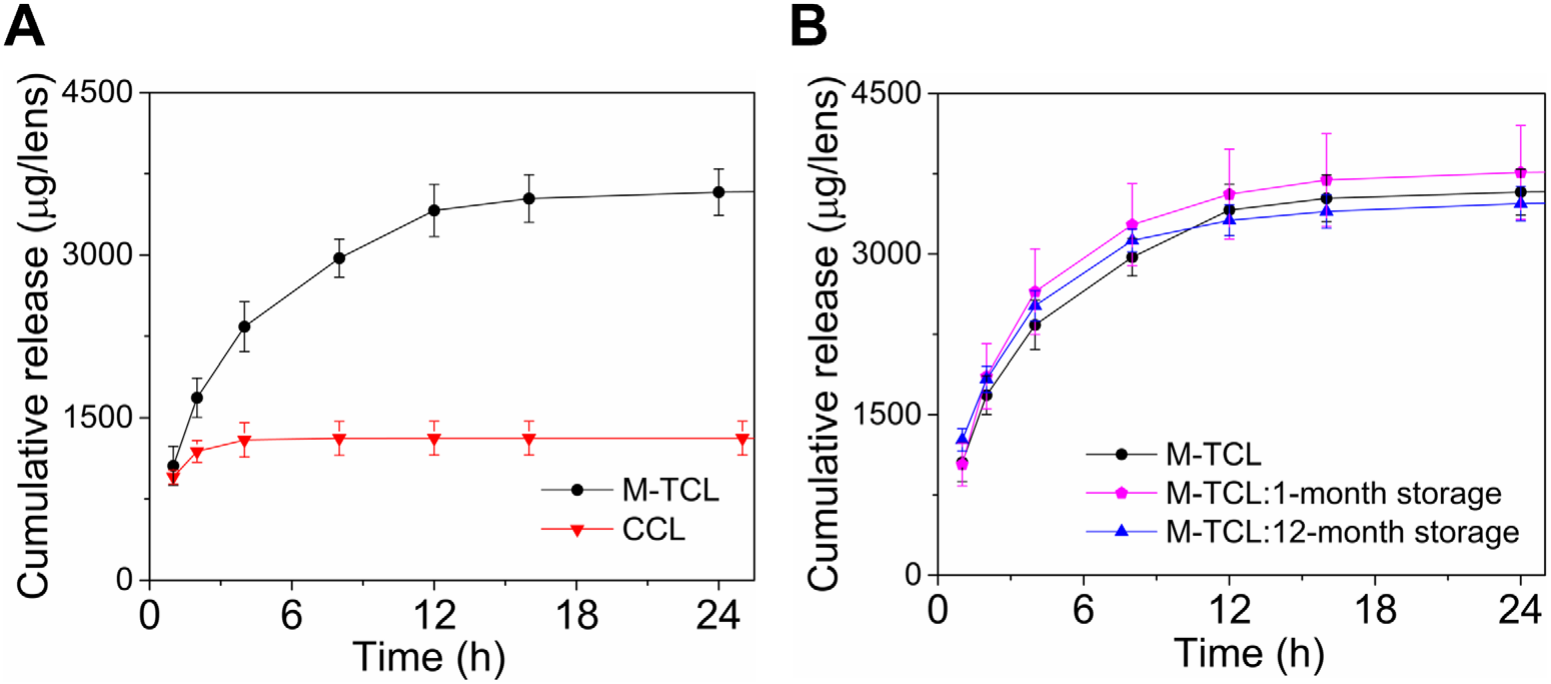
In vitro cumulative release and storage stability of M-TCLs. **(A)** Cumulative mass of moxifloxacin released from M-TCLs compared to uptake and release from commercially available Kontur CCLs. Both lens types were hydrated in a storage solution (moxifloxacin solution, 5.8 mg/mL, pH 7.4) for 24 hours. **(B)** Cumulative mass of moxifloxacin released from M-TCLs after storage for one month and 12 months in the storage solution at room temperature. Data are presented as mean ± SD (*n* = 4-5 per group per time point).

In vitro release of moxifloxacin was evaluated by removing M-TCLs from the storage solution and placing them in 5 mL of PBS at 37°C under continuous agitation. The release medium was changed at 9 predetermined time points over 48 hours and was quantified by HPLC. The in vitro release profile of hydrated M-TCL showed biphasic release behavior: (1) an initial early rapid release phase of 1055 ± 180 µg (29.2% ± 4.5%) in the first hour, likely because of, in part, the rapid release of the antibiotic that was absorbed from the storage solution; (2) a sustained release phase with a cumulative release of 3580 ± 214 µg (98.5% ± 0.7%) of moxifloxacin at 24 hours, maintaining a release rate above 8.6 µg/ h from two to 24 hours (Fig. 3A).

To better understand how the drug uptake by the hydrogel body of the contact lens influenced its drug release profile, we studied the in vitro uptake-and-release of CCLs, which had the same diameter as M-TCLs. CCLs were soaked in moxifloxacin solution and then subjected to release under the same condition as M-TCLs. CCLs released 954 ± 64 µg (73.1% ± 5.2%) of the drug within the first one hour and 1296 ± 158 µg (98.8% ± 0.5%) within the first four hours. The release rate declined to 0.3-3.6 µg/h between eight and 12 hours, and no drug was detected after 12 hours (Fig. 3A). This rapid burst release profile was consistent with previous FQ uptake-and-release studies.^32^ Given the markedly inferior release performance of CCLs (Fig. 3A) and that prior publications reported that a rapid burst release alone from therapeutic contact lenses did not reduce bacterial load in animal models of bacterial keratitis,^29^ CCLs were not pursued further for in vivo efficacy evaluation.

Premature drug release or degradation during storage could impair the effectiveness of drug-eluting contact lenses. To investigate the storage stability of M-TCLs, lenses (*n* = 4-5 per group) were stored in the moxifloxacin buffered solution for up to 12 months at room temperature. The in vitro drug release of stored M-TCLs was evaluated under the same conditions described in the section on in vitro release and storage stability study. HPLC chromatograms confirmed that the drug released from stored M-TCLs exhibited the same retention time as pharmaceutical-grade moxifloxacin, indicating no drug degradation (Supplementary Fig. S1). Furthermore, M-TCLs stored for one month and 12 months demonstrated similar total cumulative release amounts (*P* = 0.21, ANOVA) and similar release profiles with similarity factor (*f*_2_) values of 66.8 and 78.4, respectively (Fig. 3B), compared to lenses with just 24 hours’ hydration in the storage solution. According to guidelines from the Food and Drug Administration, an *f*_2_ value ≥ 50 signifies a similarity threshold, corresponding to an average difference of ≤10% at all sampling time points.^53^ In addition, all lenses maintained their morphology throughout the 12-month storage period. Unlike our previous studies on non-antibiotic drug-eluting contact lenses^8^^,^^40^^–^^44^ that used a degradable polymer, requiring dry storage and pre-hydration in a pharmacy or doctor's office before application,^28^ this system enables wet storage for up to 12 months, keeping the lenses hydrated and ready for immediate, on-demand use.

### Pharmacokinetic (PK) Profile

Ocular drug flux from M-TCLs was compared with that of 0.5% moxifloxacin ophthalmic solution (eye drops) in the eyes of New Zealand White rabbits, which have a similar eye size to human's eye.^65^ Using only the right eye, the rabbits were treated with either an intense regimen of commercial 0.5% moxifloxacin eye drops that was dosed once every 15 minutes for an hour or M-TCLs worn continuously for up to 48 hours. The intense dosing regimen of moxifloxacin eye drops, every 15 minutes initially and hourly thereafter throughout day and night,^1^ has been used as a loading dose for the treatment of severe bacterial keratitis, such as corneal ulcers.^11^ In the PK study, the administration of eye drops was stopped after the fourth dose because prior studies found that ocular drug levels plateaued at the maximum drug levels after three to four drops.^46^^,^^65^ The drug concentrations in the obtained samples of aqueous humor, cornea, other ocular tissues, and blood serum were quantified by HPLC.

#### Aqueous Humor Moxifloxacin Concentration

**Figure 4.**
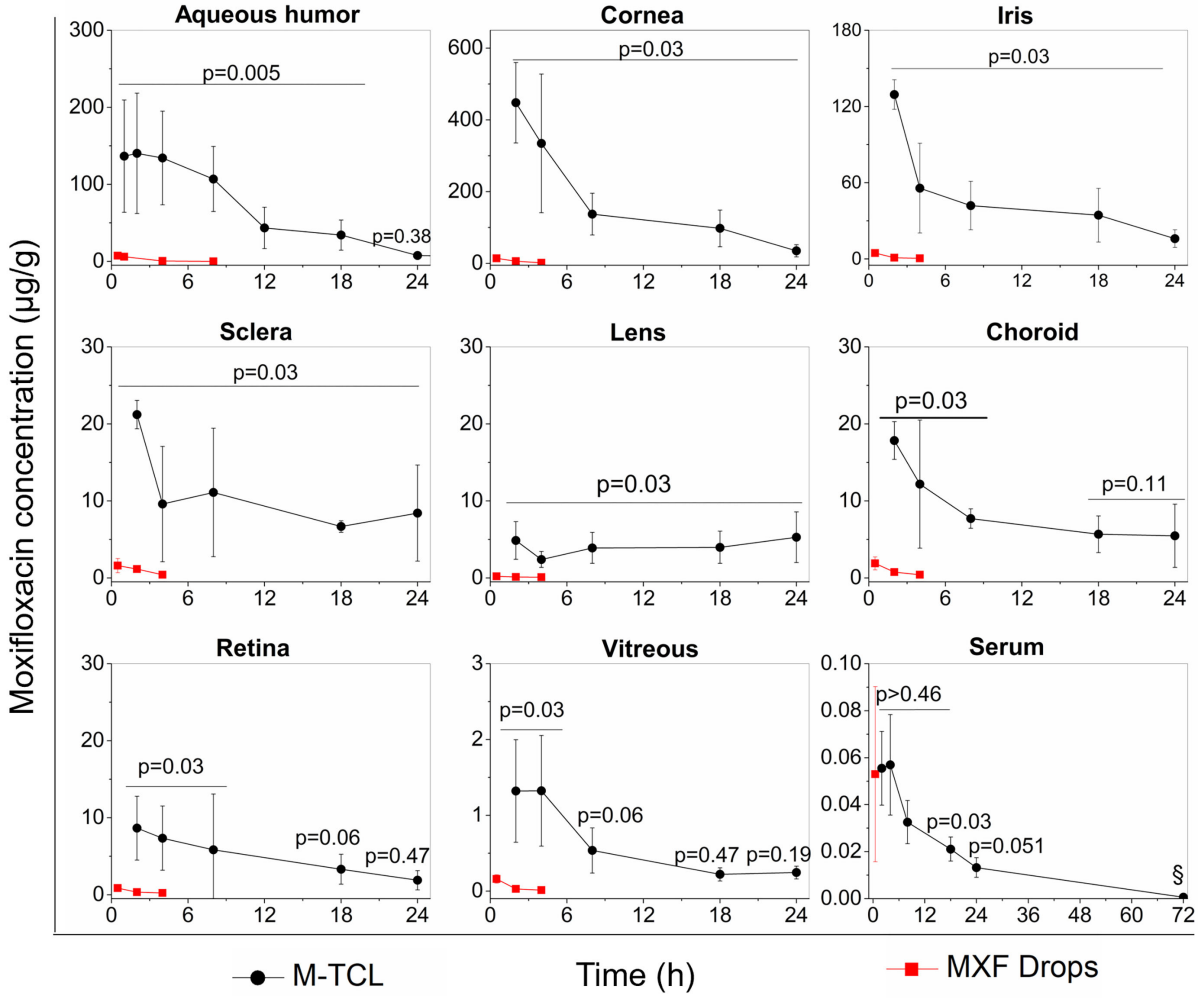
Concentrations of moxifloxacin in various ocular tissues and serum of rabbits wearing the M-TCL or receiving commercial 0.5% MXF eye drops (every 15 minutes for one hour). *P* values were calculated using Mann-Whitney U analysis to compare the drug concentrations delivered by M-TCLs at different time points with the peak concentration from MXF drops that was obtained at 0.5 hour. Data are presented as mean ± SD, *n* = 3–6 per time point per treatment. **§ =** below the lower limit of quantitation (1.5 ng/mL) for two of three animals.

After the administration of moxifloxacin eye drops or M-TCLs, the aqueous humor was removed through a 30-gauge needle inserted into the peripheral cornea of anesthetized animals. After moxifloxacin eye drop administration, the aqueous humor drug concentration peaked (C_max_) after 30 minutes at 7.5 ± 1.5 µg/mL and then declined rapidly thereafter. After administration of M-TCLs, the concentrations were the highest during the initial four hours, with a peak concentration of 140.2 ± 78.1 µg/mL measured two hours after M-TCL insertion. Compared to the intensive use of moxifloxacin drops, M-TCLs delivered significantly greater moxifloxacin concentrations to the aqueous humor. M-TCLs achieved concentrations that surpassed the peak concentration achieved by the moxifloxacin eye drops therapy for 18 hours (*P* = 0.005, *n* = 6 per time point per treatment, Mann-Whitney U, Fig. 4) and a similar concentration to C_max_ of moxifloxacin eye drops at 24 hours (*P* = 0.38).

#### Ocular Tissue Moxifloxacin Concentrations

To quantify the concentrations of moxifloxacin in ocular tissues following the treatment with moxifloxacin drops or M-TCLs, the animals were euthanized, and the tissues (*n* = 4 per time point per treatment) were collected at time points predetermined based on the aqueous humor drug flux studies (Fig. 4). Of the ocular tissues, moxifloxacin eye drops and M-TCLs delivered the highest drug concentrations to the cornea, followed by the aqueous humor > iris > sclera ≈ choroid > retina > lens > vitreous humor (Fig. 4 and Table 1). After commercial moxifloxacin eye drop administration, corneal concentrations peaked at 30 minutes (13.9 ± 0.7 µg/g) and quickly declined at the later time points (Fig. 4); these levels and drug flux pattern were consistent with previously reported studies.^27^^,^^46^^,^^66^ In contrast, after M-TCL placement, corneal concentrations peaked after two hours at 448.1 ± 112.1 µg/g, which was 32 times higher than the C_max_ for moxifloxacin eye drops (*P* = 0.03). For all the time points tested over 24 hours, the M-TCLs maintained corneal concentrations that were above the peak of the drop concentrations (*P* = 0.03, Mann-Whitney U). In addition to the cornea, M-TCLs provided significantly higher levels of moxifloxacin to the other anterior segment tissues of the eye, including the iris, sclera, and lens, for up to 24 hours (*P* = 0.03, Mann-Whitney U, Fig. 4), as well as to retina and choroid, which are posterior segment tissues, for up to eight hours (*P* = 0.03, Mann-Whitney U, Fig. 4), compared to the peak drug tissue concentrations achieved by moxifloxacin drops.

**Table 1.** M-TCLs Achieved Superior Pharmacokinetic Parameters in Ocular Tissues Compared to MXF Eye Drops (0.5%) Administered Every 15 Minutes for One Hour

Tissues	Treatment	C_max_ (µg/g)	AUC_(0–24 h)_ (µg × h/g)^*^
Aqueous humor	MXF Drops	7.5 ± 1.5	179.8^*^
	M-TCL	140.2 ± 78.1	1622.4
Cornea	MXF Drops	13.9 ± 0.7	334.6^*^
	M-TCL	448 .1 ± 112.1	3776.6
Iris	MXF Drops	4.6 ± 1.7	110.4^*^
	M-TCL	129.4 ± 11.7	1042.7
Sclera	MXF Drops	1.6 ± 0.9	38.4^*^
	M-TCL	21.2 ± 1.8	227.5
Lens	MXF Drops	0.2 ± 0.2	4.8^*^
	M-TCL	4.9 ± 2.4	92.0
Choroid	MXF Drops	1.9 ± 0.9	45.6^*^
	M-TCL	17.9 ± 2.4	188.1
Retina	MXF Drops	0.9 ± 0.4	21.6^*^
	M-TCL	8.7 ± 4.2	112.4
Vitreous humor	MXF Drops	0.2 ± 0.06	3.8^*^
	M-TCL	1.3 ± 0.7	12.9

*n* = 4–6 per time point per treatment. The maximum concentrations (C_max_) data are expressed as mean ± standard deviation. The area under the concentration time curve from 0 to 24 hours (AUC_(0–24 h)_) for M-TCL was calculated using linear trapezoid rules.

^*^AUC_(0–24 h)_ for moxifloxacin drops was overestimated to be 24 times C_max_ by assuming continuous administration of moxifloxacin drops every 15 minutes for 24 hours (total 96 drops), which is not feasible in clinical practice yet.

#### Blood Serum Moxifloxacin Concentration

The systemic drug exposure was evaluated by quantifying moxifloxacin levels in the blood serum of rabbits after treatment with moxifloxacin drops or M-TCLs. The serum drug levels 0.5 hour were measured after administration of four drops of moxifloxacin, because this was the T_max_ (time until maximum concentration) reported by previous studies.^11^ The serum drug level following drop administration at 0.5 hour was 53.0 ± 37.3 ng/mL, which is comparable to previously reported C_max_ values.^11^ Since the T_max_ for M-TCLs was unknown, blood serum concentrations after the application of M-TCLs were measured throughout the ocular pharmacokinetic study, with an additional measurement at 72 hours to assess systemic absorbance. The C_max_ of M-TCL in blood serum occurred at 4 hours (57.0 ± 21.4 ng/mL), which was similar to that of moxifloxacin drops at 0.5 hour (*P* = 0.47, Mann-Whitney U, Fig. 4). The blood serum levels declined rapidly 4 hours after M-TCL placement and were undetectable in two of three serum samples after 72 hours. These findings indicate that M-TCLs resulted in systemic drug exposure comparable to traditional moxifloxacin drops therapy.

### In Vivo Efficacy Against MRSA Keratitis in a Rabbit Model

**Figure 5.**
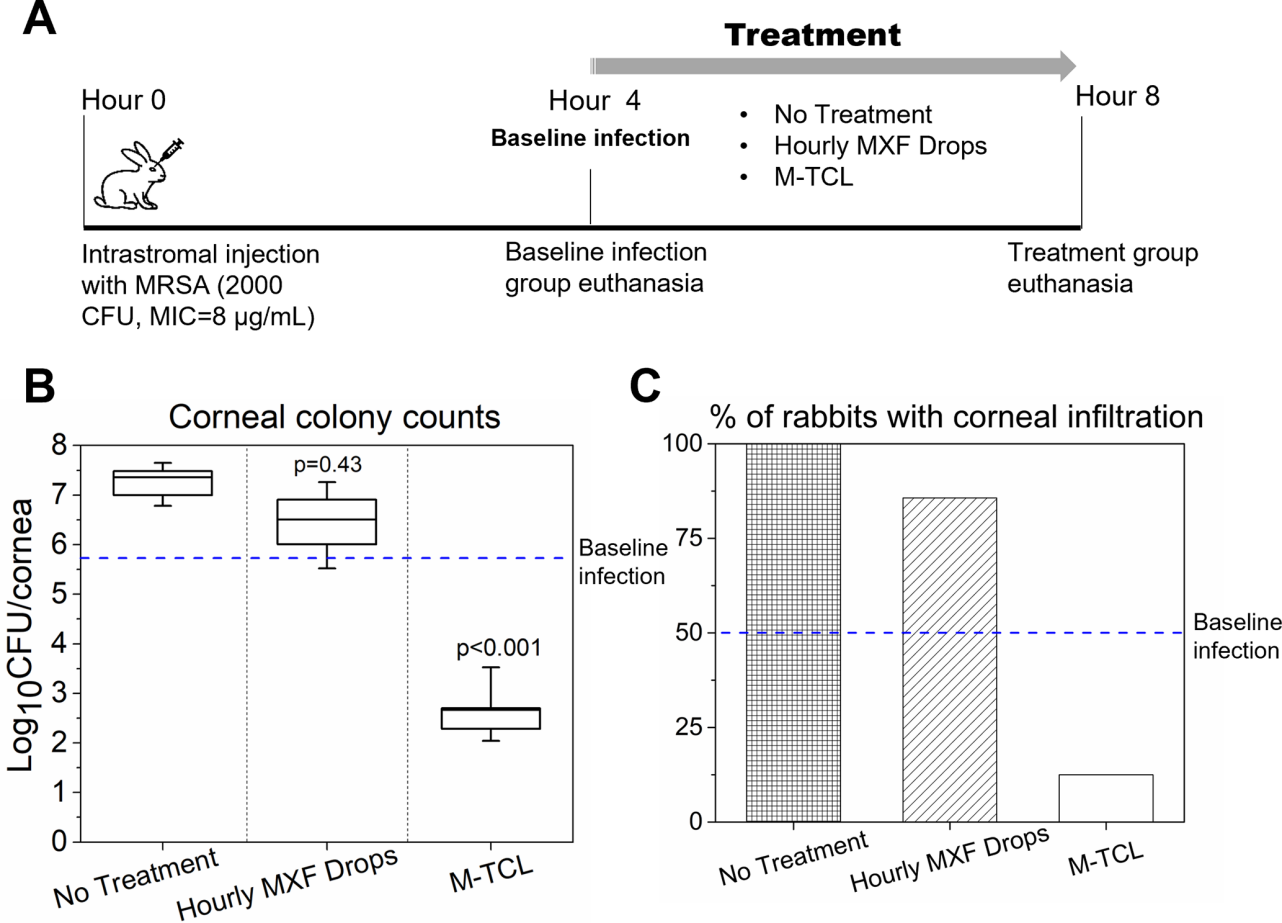
M-TCLs significantly reduced bacterial loads in a rabbit model of MRSA keratitis. **(A)** Schematic illustration of animal experiment design. **(B)** Box plots of MRSA CFUs in the infected rabbit eyes after no treatment, or treatment with hourly 0.5% MXF drops or an M-TCL. The resulting CFUs after treatments were compared with that of the no treatment group using a one-way ANOVA followed by Tukey post hoc. The *blue dashed line*, *n* = 7, represents the bacterial load at baseline infection (the onset of treatment). M-TCLs (*n* = 8) significantly reduced the CFU of MRSA in the infected eyes, compared with hourly MXF drops (*n* = 7) (*P* < 0.001, Tukey). **(C)** Bar plots of percentage of the infected eyes with corneal infiltration after different treatments.

To test whether the superior corneal drug flux from M-TCLs translated into greater treatment effectiveness, in vivo efficacy of M-TCLs was evaluated using a well-established keratitis model in rabbits^3^^,^^56^^,^^57^ and compared with that of hourly moxifloxacin drops—a commonly used intensive regimen in current clinical practice.^67^^,^^68^ To determine whether M-TCLs can overcome bacterial resistance, we chose an MRSA strain (MIC = 8 µg/mL) that was considered resistant to moxifloxacin according to the CLSI standards that have been established for systemic blood serum.^55^ CLSI guidelines for systemic antibiotics were used instead, because the standard for topical antibiotics is still lacking.^3^^,^^56^^,^^57^ MRSA keratitis was established through an injection under an intact corneal epithelium into the corneal stroma using an MRSA inoculum of 25 µL and 80 CFU/µL (Fig. 5A).

#### Bacterial Count

After corneal intrastromal injection and four hours of intraocular incubation to allow the progression and establishment of the infection before treatment, a randomized group (*n* = 7) of rabbits was euthanized to determine the baseline corneal bacterial loads (i.e., baseline infection). The remaining rabbits were randomized into one of three treatment groups: (1) no treatment (*n* = 7), (2) hourly commercial moxifloxacin 0.5% eye drops (*n* = 7), or (3) a single M-TCL (*n* = 8). The corneal bacterial count in this baseline infection group was measured at 5.76 ± 0.63 log_10_ CFU/cornea (*n* = 8), confirming the establishment of MRSA keratitis.^3^ In the no treatment group, the bacterial load *increased* by 1.48 log-units compared to the baseline infection (*P* < 0.0001, Fig. 5B), consistent with previous studies.^56^ The hourly moxifloxacin eye drops group exhibited 0.71 log-unit *greater* CFUs than the baseline infection group (*P* = 0.049). The difference in bacterial burden between the no treatment group and moxifloxacin eye drops group was not statistically significant (*P* = 0.43). In contrast to the progression of the infection in the eye drops group, the M-TCL group resulted in a 3.15 log-unit *reduction* in bacterial load compared to the baseline infection group, which equated to a 99.95% killing of the MRSA. In addition, the resulting CFUs were 4.63 log-units lower than those in the no treatment group (*P* < 0.0001) and 3.86 log-units lower than those in the moxifloxacin drops group (*P* < 0.0001, Fig. 5B).

#### Drug Flux in Keratitis Rabbits

In addition to the PK study in healthy rabbits—where concentrations achieved with M-TCL were compared to the maximum concentration obtained via an intensive moxifloxacin drops, additional drug flux was also investigated in rabbits with keratitis. Briefly, moxifloxacin corneal concentrations in keratitis rabbits were quantified at the time of euthanization, which occurred approximately one hour after the last moxifloxacin drop or about four hours after M-TCL insertion. The moxifloxacin corneal concentrations (5.8 ± 2.5 µg/g, *n* = 7) from hourly drops were within the range of the concentration from the pharmacokinetic study in healthy rabbits that used drops applied every 15 minutes (6.1 ± 1.4 µg/g, *n* = 4, Fig. 4). These findings align with prior studies showing that repeated eye drop instillations quickly reach a concentration plateau in the cornea, with hourly administration maintaining that plateau.^46^^,^^65^ In contrast, the corneal moxifloxacin concentration (350.0 ± 86.9 µg/g, *n* = 6) in M-TCL-treated keratitis rabbits were significantly higher than in drop-treated keratitis rabbits (350.0 ± 86.9 µg/g vs. 5.8 ± 2.5 µg/g, *P* = 0.0012), and closely matched the concentration in healthy rabbits wearing M-TCL for the same duration (four hours) in the PK study (334.5 ± 193.2 µg/g, *P* = 1; Fig. 4).

#### Sustained Effect of M-TCL

To evaluate whether the efficacy of M-TCL could be sustained beyond the four-hour treatment window of the adopted animal model, additional experiments were conducted using M-TCLs that had been pre-worn by healthy rabbits for eight hours (M-TCL_preworn8h_) or 18 hours (M-TCL_preworn18h_) (*n* = 4 per group). The pre-worn M-TCLs were then tested using the same keratitis model described previously, maintaining consistent incubation and treatment periods of four hours each. The M-TCL_preworn8h_ resulted in 2.70 log-unit reduction in CFUs compared to the baseline infection (*P* = 0.013), which corresponds to 99.8% bacterial killing. The resulting CFUs per cornea were 3.69 log-unit lower than those in the no treatment group (*P* < 0.0001; Table 2). The M-TCL_preworn18h_ resulted in a modest reduction of 0.58 log units in CFUs compared to baseline infection (*P* = 0.80). The CFUs per cornea were 2.12 log-units lower than those in the no treatment group (*P* = 0.003; Table 2). These findings demonstrated the sustained effectiveness of M-TCLs in reducing bacterial burden, even when the lenses were pre-worn for extended periods.

**Table 2.** M-TCLs (*n* = 4–8 per Group) Significantly Reduced Bacterial Growth to a Greater Extent Than Hourly 0.5% MXF (*n* = 7) Eye Drops in the Rabbit Model MRSA Keratitis

Treatment Group	Log (CFU)	*P* Value Compared With Hourly MXF Drops	*P* Value Compared With No Treatment
No treatment	7.24 ± 0.31	0.43	NA
Hourly MXF drops	6.47 ± 0.57	NA	0.43
M-TCL_preworn0h_	2.61 ± 0.45	<0.001	<0.001
M-TCL_preworn8h_	3.55 ± 1.88	<0.001	<0.001
M-TCL_preworn18h_	5.12 ± 1.57	0.1	0.003

M-TCL_0h_, treatment with an M-TCL that has been pre-worn by a healthy rabbit for zero hours before being placed on infected eyes; M-TCL_8h_ treatment with an M-TCL that has been pre-worn by a healthy rabbit for eight hours before being placed on infected eyes; M-TCL_18_, treatment with an M-TCL that has been pre-worn by a healthy rabbit for 18 hours before being placed on infected eyes.

Log (CFU) data is expressed as mean ± standard deviation. *P* values from a one-way ANOVA followed by Tukey post hoc.

#### Anti-Inflammatory Effects

##### Aqueous Humor Protein Levels

Bacterial keratitis is typically accompanied by an inflammatory response in the aqueous humor, and generally, the severity of the infection is correlated with the intensity of this inflammation.^69^^,^^70^ We used a Bradford Assay to quantify the concentration of aqueous humor protein, which is a biomarker for intraocular inflammation.^8^^,^^44^^,^^71^

**Figure 6.**
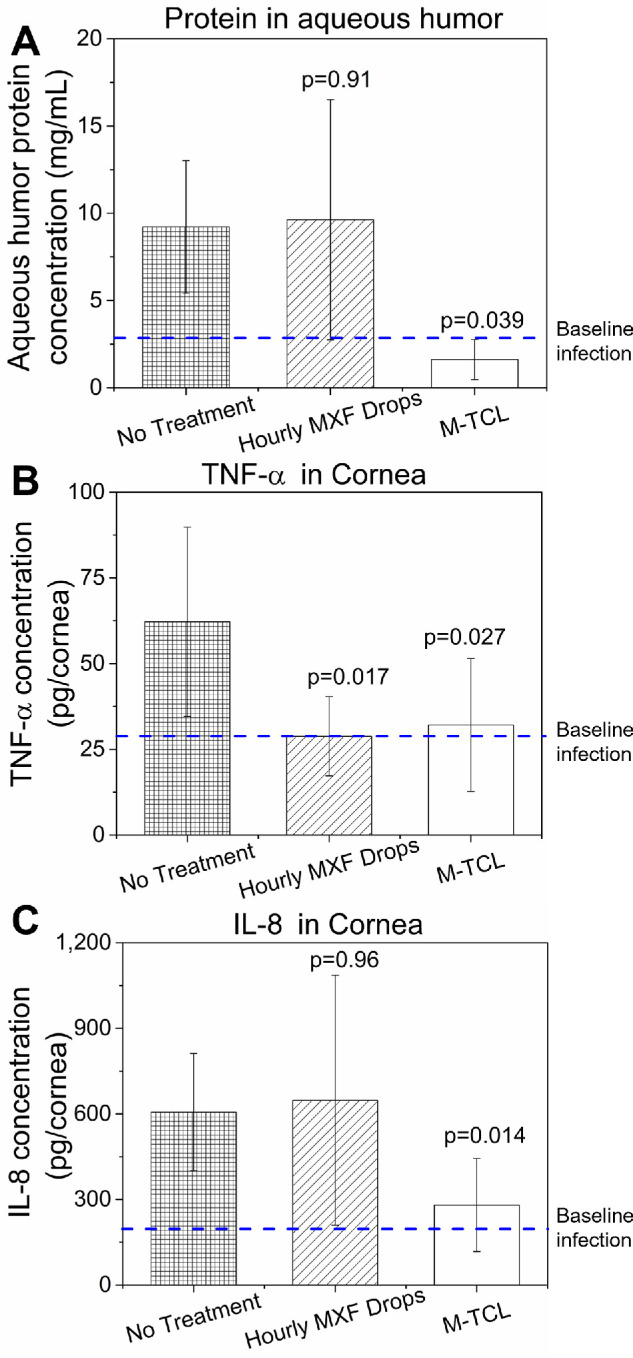
Inflammatory biomarkers in MRSA-infected eyes after no treatment, or treatment with hourly 0.5% MXF drops or M-TCL. **(A)** Aqueous humor protein levels, **(B)** TNF-α expressions, and **(C)** IL-8 expressions after different treatments. The *blue dashed line* represents the relative level at the baseline infection. *P* values as compared with no treatment using a one-way ANOVA followed by Tukey post hoc. Data are presented as mean ± SD, *n* = 4–8 per group.

The protein level in the aqueous humor in the uninfected contralateral eyes (*n* = 11) was 0.54 ± 0.42 mg/mL, consistent with previous studies by our team^44^ and other groups.^72^^,^^73^ Four hours after the intrastromal injection of the bacterial inoculum, the aqueous humor protein concentration in the baseline infection group (*n* = 7) experienced a 5.2-fold increase to 2.81 ± 3.29 mg/mL, indicating the presence of an inflammatory response to bacterial keratitis.^70^ The aqueous humor protein level in the no treatment group was found to be 3.3-fold higher than the baseline infection group (*P* = 0.032; Fig. 6A), indicating that the intraocular inflammatory response increased with time in the absence of antibiotics. The hourly moxifloxacin eye drops group showed a similar aqueous humor protein level compared to the no treatment group (*P* = 0.91). In contrast, the M-TCL group (*n* = 8) resulted in a significantly lower aqueous humor protein level, 82.4 % lower than the no treatment group (*n* = 4; *P* = 0.039) or 83.2 % lower than the hourly moxifloxacin drops group (*n* = 7; *P* = 0.01).

##### Corneal Pro-Inflammatory Cytokine Levels

Using ELISA assays, we measured the corneal levels of TNF-α and IL-8, which are pro-inflammatory cytokines associated with corneal tissue damage resulting from bacterial infections.^74^ The expression of TNF-α and IL-8 in the cornea was undetectable in the uninfected, contralateral eyes. In the baseline infection group (*n* = 6), the TNF-α concentration was 28.9 ± 12.4 pg/cornea and IL-8 concentration was 196.6 ± 80.5 pg/cornea; these enhanced levels confirmed the presence of an inflammatory response to the induction of bacterial keratitis.^75^ Compared to the baseline infection, the no treatment group (*n* = 7) experienced a 2.2-fold increase in TNF-α expression (62.2 ± 27.6 pg/cornea, *P* = 0.019) and a 3.1-fold increase in IL-8 levels (606.7 ± 205.6 pg/cornea, p = 0.006). The TNF-α concentrations in eyes treated with hourly moxifloxacin drops (*P* = 0.017, *n* = 7) and M-TCLs (*P* = 0.027, *n* = 8) were both significantly lower than the no treatment group (Fig**.** 6B). For IL-8, hourly moxifloxacin eye drops group (*n* = 7) showed similar expression compared to the no treatment group (*n* = 7; *P* = 0.96), whereas M-TCLs (*n* = 8) successfully suppressed IL-8 expression compared to both the no treatment group (*P* = 0.014) and the hourly moxifloxacin eye drops group (*P* = 0.13; Fig. 6C).

#### Clinical Observations

Two masked ophthalmologists graded clinical signs of infection from photographs of the study eyes. An established scoring system with a grading scale ranging from zero to four was employed to assess the severity of conjunctival redness, conjunctival edema (chemosis), and corneal infiltration. A score of zero denotes normal findings and higher grades indicate a progressively more severe infection.^60^

The baseline infection group (*n* = 8) had a total clinical score of 2.5 ± 0.6. The total clinical score of the no treatment group (*n* = 7) was 4.5 ± 1.3, which was 1.6-fold higher than the baseline infection (*P* = 0.025, Mann-Whitney U test). Consistent with previous reports, animals treated with hourly moxifloxacin eye drops (*n* = 7) had a similar total clinical score (4.0 ± 1.7) to the no treatment group.^76^ Rabbits treated with M-TCLs (*n* = 8) resulted in a lower total clinical score (2.3 ± 1.4) that approached statistical significance when compared to the no treatment or hourly moxifloxacin drops (*P* = 0.09, Kruskal-Wallis ANOVA).

The amount of corneal infiltration is correlated with the severity of infection, subsequent scarring, and impairment of vision.^69^ In this study, up to 50% of the eyes in the baseline infection group and 100% of the eyes in the no treatment group had corneal infiltration graded ≥1 (Fig. 5C). Eighty-six percent of infected eyes treated with hourly moxifloxacin eye drops had corneal infiltration scores ≥1. In contrast, only very light corneal infiltration was observed in one out of eight (12.5%) of the infected eyes after the M-TCL treatment. The remaining seven infected eyes had no infiltration after M-TCL treatment.

### In Vivo Irritation and Biocompatibility Study

#### Ocular Irritation Study (Draize Test)

**Figure 7.**
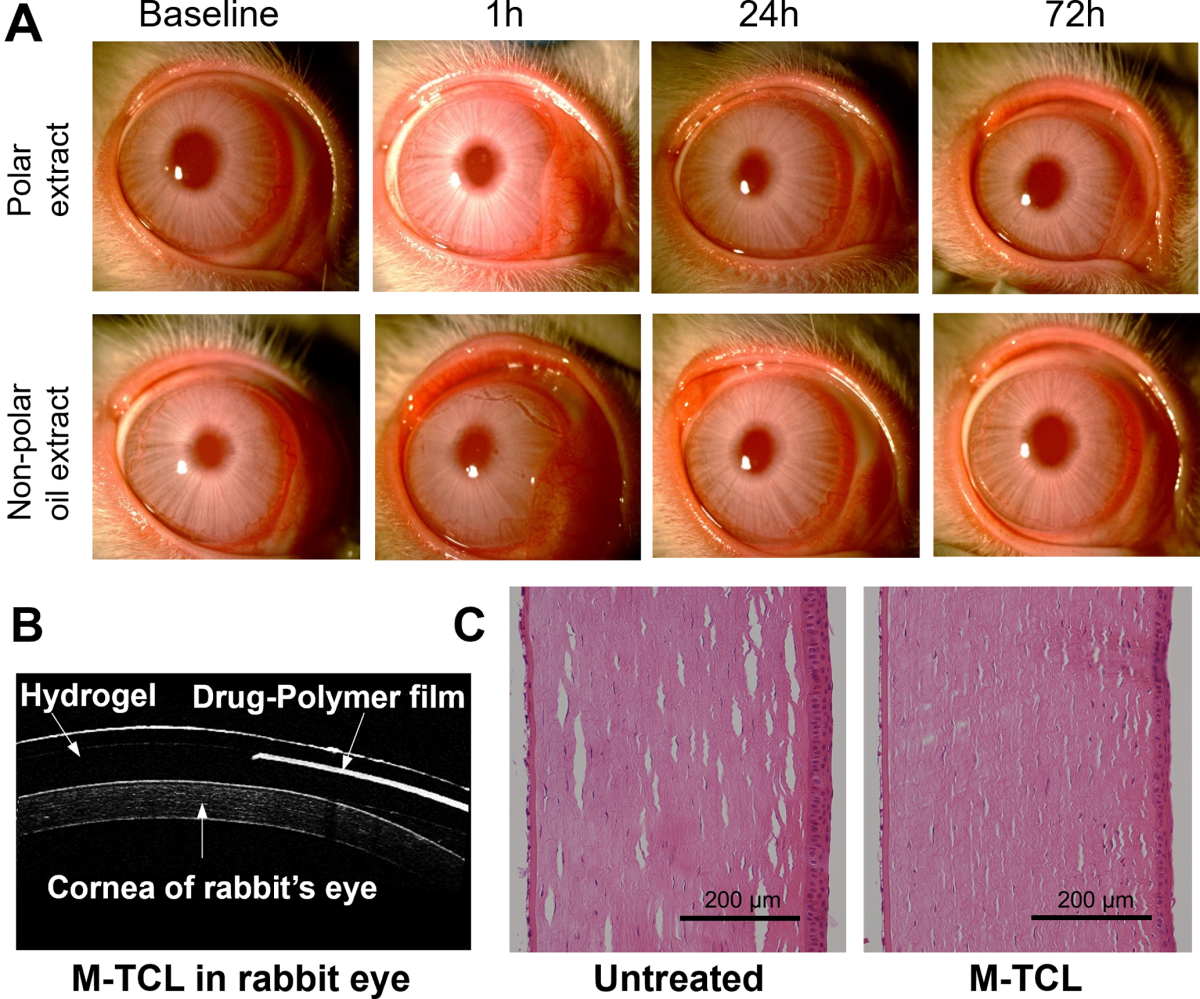
In vivo biocompatibility studies of the M-TCL in a healthy rabbit model. M-TCLs did not introduce ocular irritation, inflammation, or any other pathology, as assessed by slit lamp biomicroscopy, OCT, and histology analysis. **(A)** Representative slit lamp photographs of study eyes at baseline and one, 24, and 72 hours after the instillation of either polar extract (in sterile saline solution, *n* = 3) or non-polar extract (in cottonseed oil, *n* = 3) from the M-TCL. **(B)** OCT side-view image of the normal rabbit cornea with the M-TCL in place. **(C)** Representative H&E-stained histology image of the cornea (*n* = 3) after 72 hours of continuous M-TCL wear. The untreated contralateral (normal) eye served as a control.

Draize test is commonly used and endorsed by the Food and Drug Administration as an indirect method to determine the biocompatibility and irritation potential of leachable materials or pharmaceuticals that come into contact with the eyes.^61^ In this study, the Draize test was performed according to OECD standards. Briefly, M-TCLs were extracted for 72 hours by either a polar solvent (sterile saline) or a non-polar solvent (cottonseed oil). Then, the extract was instilled in the test eye of the rabbits (*n* = 3). After extract instillation, the rabbit eyes were examined by slit lamp biomicroscopy and graded by an ophthalmologist at one, 24, and 72 hours using the OECD grading scale.^44^ No corneal or conjunctival lesions or abnormalities were observed during the study. The scores of treated eyes were identical to the baseline or untreated (normal) contralateral eyes at all the time points, demonstrating that both polar and non-polar extracts from M-TCL did not induce any ocular irritation (Fig. 7A).

#### Histology

The direct biocompatibility of M-TCLs worn continuously by rabbits for 72 hours was also evaluated. The rabbits were regularly monitored for signs of discomfort, including ocular redness, lid edema, and other corneal complications. No evident signs of discomfort or adverse effects were observed. Of note, the M-TCL retained its normal shape throughout the 72-hour wear period. OCT images of the M-TCL on the rabbit eye, captured at the end of the wear period, confirmed the lens's morphology, showing a homogenous drug-polymer film fully encapsulated within the methafilcon hydrogel and a clear central aperture for unobstructed vision (Fig**. 7**B).

After 72 hours, the rabbits were euthanized, and their eyes were enucleated for histological analysis. H&E-stained histology slides, assessed by ophthalmologists, revealed that corneas of the M-TCL group were indistinguishable from those of the untreated (normal) contralateral eyes (Fig. **7**C). The corneal tissue remained structurally intact, with a normal epithelium, stroma, and endothelium, and no signs of inflammatory cell infiltration. The epithelial layer consisted of three to four layers of regularly arranged squamous cells. These findings indicated that the M-TCL was biocompatible and safe in the rabbit eye.

## Discussion

This study reports the study of moxifloxacin-eluting therapeutic contact lens (M-TCL) for improved treatment of bacterial keratitis. The M-TCL contained a ring-shaped antibiotic-polymer film encapsulated within the periphery of the methafilcon hydrogel body, forming a central aperture for unimpeded vision (Fig. 1). Methafilcon hydrogel, a material widely used in commercial soft contact lenses, provides a biocompatible and comfortable substrate. Using Ethocel ethylcellulose, a physiologically inert and biocompatible polymer,^62^ in the drug-polymer film matrix offered enhanced stability of the drug delivery zone. In rabbit eyes, M-TCLs maintained moxifloxacin corneal concentrations for 24 hours at levels far exceeding those achieved by intensive moxifloxacin drops (Fig. 4). Importantly, M-TCLs demonstrated potent bactericidal effects in a rabbit model of bacterial keratitis that resulted from intrastromal injection of an MRSA isolate (MIC = 8 µg/mL), which exhibited in vitro resistance to moxifloxacin according to CLSI systemic blood serum guidelines. In contrast, hourly moxifloxacin eye drops failed to reduce the bacterial load from baseline infection (Fig. 5B). M-TCL efficacy was further evidenced by reductions in inflammatory biomarkers and clinical signs (Figs. 5C, 6), highlighting its potential as a superior treatment option for drug-resistant bacterial keratitis.

M-TCLs demonstrated key physical properties within the typical range reported for commercial contact lens materials, including morphology, central light transmittance (94.2% ± 0.3% vs. 90%–100%^45^), water content (54.4% ± 6.4% vs. 38%–74%^45^^,^^52^), and mechanical strength (0.50 ± 0.05 MPa vs. 0.3-0.6 MPa^52^). Although these findings confirm the overall suitability of M-TCLs for ocular applications, light transmittance warrants particular attention because it directly affects visual performance.^45^ The drug–polymer film region remained semitransparent (82.4% ± 2.0% transmittance), consistent with cosmetic colored lenses (55%–85%).^8^^,^^77^ Although lens movement could theoretically shift peripheral regions of the drug–polymer film toward the visual axis, such displacement is expected to be minor (0.2–0.4 mm, lag method) and transient.^78^^,^^79^ Moreover, the clear central aperture (5.2 mm) exceeds typical pupil diameters under photopic (3.0–4.5 mm) and most mesopic (4.5–6.5 mm) conditions,^80^ whereas the semitransparent film occupies only the periphery, similar to the tinted regions of cosmetic lenses. Collectively, these factors indicate that any potential reduction in visual performance is expected to be minimal and clinically insignificant.

The global overuse of antibiotics has led to a growing public health crisis of drug-resistant infections. Resistance is more likely to develop when drug concentrations fall below the mutant prevention concentration, the threshold needed to inhibit the growth of “first-step” resistant mutants.^81^ As antibiotic-resistant bacteria are evolving faster than new antibiotics are developed, sustained drug delivery systems have garnered increasing attention.^71^^,^^82^^–^^85^ We hypothesized that sustained drug delivery could overcome resistance in bacterial keratitis, as in other diseases, by providing the cornea with sustained antibiotic delivery at concentrations that far exceed MIC. This strategy aligns well with FQ antibiotics, whose efficacy depends on both achieving drug concentrations above MIC and maintaining these levels for an adequate duration.^11^ Using moxifloxacin, a widely used FQ antibiotic, the M-TCL exemplifies this approach by successfully overcoming resistance in bacterial keratitis. The M-TCL has two-phases exhibited with the in vitro release profile: an initial early rapid release phase within the first few hours to rapidly reach therapeutic concentrations, followed by a sustained release phase to maintain these levels over time—an advantage not seen with commercial soak-and-release contact lenses (Fig. 3). In vivo, M-TCLs quickly attained C_max_ of 448.1 ± 112.1 µg/g, well above the MRSA MIC (8 µg/mL) within the initial early rapid release phase, and maintained levels above MIC for 24 hours in the cornea during the sustained release phase. In contrast, moxifloxacin eye drops, which failed to treat MRSA keratitis, resulted in a much lower corneal C_max_ of 13.9 ± 0.7 µg/g, with concentrations declining rapidly after reaching the peak. The area under the concentration-time curve (AUC), a standard metric for drug bioavailability,^41^ further underscores the advantages of M-TCLs. The corneal AUC_(0–24 h)_ for M-TCL, calculated using the trapezoidal method, was 3776.6 µg × h/g (Table 1). Even under an extreme and impractical scenario of administering moxifloxacin eye drops every 15 minutes for 24 hours (96 drops, totaling 14,400 µg of moxifloxacin),^86^ the AUC_(0–24 h)_ for eye drops was only 334.6 µg × hours/g, 11.3-fold lower than that of M-TCLs. Comprehensive comparisons of C_max_ and AUC across all tested tissues (Table 1) further highlight the pharmacokinetic superiority of M-TCLs, supporting their efficacy in treating MRSA keratitis. Despite achieving high drug concentrations, the C_max_ attained by M-TCLs remained below reported cytotoxicity thresholds for human or rabbit corneal endothelial and epithelial cells.^87^^–^^90^ Furthermore, M-TCLs were safe in healthy rabbit eyes after continuous wear for 72 hours (Fig. 7).

In contrast to current treatments requiring frequent administration of topical antibiotic eye drops or ointments day and night, a single M-TCL provided sustained moxifloxacin delivery for over 24 hours, potentially improving patient compliance.^2^^,^^7^ In typical clinical scenarios where patients with vision-threatening corneal ulcers are seen daily, M-TCLs can be applied at the initial presentation of a corneal ulcer (bacterial keratitis) and replaced or removed the next day during follow-up. Moreover, the M-TCL functions as both a therapeutic agent and a non-medicated protective bandage contact lens, offering a streamlined and synergistic treatment, particularly in cases involving trauma or lagophthalmos (incomplete eyelid closure), which lead to exposure keratopathy.^2^ In addition, in cases where infections are unresponsive to traditional therapies^2^ or when patients face physical or psychological barriers to self-administer hourly eye drops and ointments,^91^ M-TCLs have the potential to reduce hospital admissions, lowering healthcare costs.

Compared to previously reported antibiotic-eluting contact lenses, M-TCLs offer unique advantages. Traditional soak-and-release approaches using commercial contact lenses often resulted in rapid drug release, with the majority of the antibiotic being released within a few hours. Efforts to improve this approach, such as incorporating diffusion barriers (e.g., polymer films, vitamin E, and nanoparticles) or employing binding techniques (e.g., molecular imprinting, ion complexes, and supercritical carbon dioxide), have greatly extended in vitro drug release durations or increased the total drug release amount.^27^^–^^39^ However, like other moxifloxacin delivery systems, few of these technologies have demonstrated both robust in vivo efficacy and favorable pharmacokinetics. Moreover, those that have been tested in vivo were generally targeted susceptible bacterial strains, with FQ MICs ranging from 0.06 µg/mL to 0.4 µg/Ml.^27^^,^^29^^,^^32^^,^^35^^,^^39^^,^^92^ For instance, using the soak-and-release method, hydroxyethyl methacrylate (HEMA) contact lenses significantly prolong the in vitro release of gatifloxacin or moxifloxacin through ionic bonding between the drug and HEMA. In a rabbit model of bacterial endophthalmitis, the gatifloxacin-soaked HEMA contact lenses significantly inhibited the proliferation of *S.* *aureus* (MIC = 0.12 µg/mL) at 24 hours.^27^ However, these lenses were evaluated for prophylactic use rather than the treatment of established infections. In another rabbit study, contact lenses embedded with semicircular rings containing moxifloxacin and hyaluronic acid showed clinical outcomes equivalent to moxifloxacin eye drops in treating *S.* *aureus* (MIC = 0.06 µg/mL) conjunctivitis—a condition that is often self-limiting.^32^ For bacterial keratitis treatment, ciprofloxacin-imprinted silicone lenses achieved approximately a 2-log CFU reduction of ciprofloxacin-susceptible *P. aeruginosa* (MIC = 0.4 µg/mL), whereas hourly ciprofloxacin eye drops completely sterilized rabbit corneas within 24 hours.^29^ Similarly, dual drug-eluting mucoadhesive contact lenses releasing moxifloxacin and dexamethasone showed only modest improvement over a single moxifloxacin drop in treating *S.* *aureus* (MIC of moxifloxacin = 0.126 ± 0.057 µg/mL) keratitis, with bacterial reduction limited to 1.2 log CFU and residual populations still around 8 log units.^35^

In contrast to previous studies, we tested M-TCLs bactericidal efficacy against MRSA keratitis caused by a resistant clinical isolate (MIC = 8 µg/mL), which is particularly challenging to treat. M-TCLs achieved a 4.63 log-unit reduction compared to no treatment group (*P* < 0.0001), and a 3.86 log-unit reduction compared to the moxifloxacin drops group (*P* < 0.0001; Fig. **5**B), underscoring the importance of maintaining sustained and elevated drug concentrations above the MIC to effectively combat antibiotic resistance. Additionally, M-TCLs provide practical benefits. When packaged in a hydrated, sterilized state, they are ready for immediate use, eliminating the need for time-consuming hydration processes. M-TCL achieved a shelf life of up to 12 months in a hydrated state (Fig. 3B), offering enhanced convenience and accessibility for both patients and healthcare providers.

M-TCLs also effectively mitigated the inflammation associated with bacterial keratitis, as evidenced by the decreased levels of aqueous humor protein and corneal cytokines (Fig. 6). The reduction in inflammation is likely attributable to both the antibacterial effect of the M-TCL and the dose-dependent anti-inflammatory effects of moxifloxacin.^76^^,^^85^ The M-TCL inhibited bacterial replication and growth, which, in turn, presumably alleviated corneal inflammation. Additionally, moxifloxacin showed the ability to reduce pro-inflammatory cytokines (e.g., IL-8, TNF-α) at concentrations exceeding 10 µg/mL, presumably via inhibition of the mitogen-activated protein kinase signaling pathways.^76^ However, more research is needed to fully elucidate its anti-inflammatory mechanisms in the corneal environment.

Although we used an established rabbit model^3^^,^^56^^,^^57^ for the efficacy study, one limitation of this study was the relatively short incubation time of four hours to establish the MRSA infection. At the onset of the treatment, the bacterial load reached 5.76 log CFU per cornea in this study. With an incubation time of nine to 15 hours, it was reported that *S. aureus* could reach a maximum bacterial load of 7-8 log CFU per cornea and generate more toxins when its growth reaches the stationary phase of the growth curve.^93^ Nevertheless, the limited incubation time in our study is partially compensated by the specific characteristics of our model. By injecting a large bacterial inoculum (2000 CFU in this study vs. 100 CFU in other rabbit models^93^) into the stroma while leaving the epithelium intact, our model replicates an established infection that is more challenging to treat when there is an infiltrate present under an intact epithelium, which acts as a barrier to drug delivery. This model also reduces animal suffering while providing a difficult-to-treat scenario. Another limitation of the study is the relatively short treatment time of four hours. Although this model is a suitable means of comparing treatments and their ability to reduce bacterial loads within the study window, we further tested the efficacy of M-TCL that had released moxifloxacin for eight and 18 hours in treating MRSA keratitis (Table 2). Specifically, after M-TCLs had been worn by healthy rabbits for either eight or 18 hours, they were removed and placed in the eyes of rabbits that had received intrastromal injection of MRSA inoculum. Compared to no treatment, the M-TCL_preworn8h_ and M-TCL_preworn18h_ groups showed 3.69 (99.99% killing; *P* < 0.0001) and 2.12 (97.50% killing; *P* = 0.003) log-unit reductions in CFUs, respectively (Table 2). These observations demonstrate that the effectiveness of M-TCL against MRSA keratitis can be extended beyond the four-hour treatment window.

The M-TCL shows promise as a convenient and effective tool to treat bacterial keratitis, including drug-resistant infections. Our study also contributes insights into overcoming antibiotic resistance by delivering sustained and elevated drug concentrations at targeted tissues that surpass the MIC. However, several avenues remain for further research. From a materials perspective, future studies are needed to elucidate the mechanism by which moxifloxacin prevents delamination. To better correlate in vitro release with therapeutic performance in infected eyes, additional in vitro release studies using M-TCLpreworn_8h_ and M-TCLpreworn_18h_ will be conducted. To further enhance the translational relevance of efficacy evaluation, future studies may consider including a loading dose of drops applied every 15 minutes followed by hourly eye drops or uptake-and-release CCLs as control groups. Clinically, additional studies, including clinical trials, are necessary to evaluate M-TCLs' long-term safety, in vivo efficacy against gram-negative bacteria (such as *P.* *aeruginosa*) and bacterial biofilms, and their potential to improve patient adherence and treatment outcomes in humans.

## Supplementary Material

Supplement 1
