# Supplementary material for: Overcoming Antimicrobial Resistance in Bacterial Keratitis With an Antibiotic-Eluting Contact Lens
Source: Invest Ophthalmol Vis Sci. 2025 Dec 12;66(15):38. doi: 10.1167/iovs.66.15.38 (PMC12707326; doi:10.1167/iovs.66.15.38)
Supplement: Supplement 1 [file iovs-66-15-38_s001.pdf]

Supporting information

Overcoming antimicrobial resistance in bacterial keratitis with an antibiotic-eluting contact lens

Liangju Kuang[1], Amy E. Ross[1], Lin Chen[1], Levi N. Kanu[1], Nikolay Boychev[1], Alireza Ghaffarieh[1], Cheng Peng[1], Paulo J. M. Bispo[2], Eric G. Romanowski[3], Daniel S. Kohane[4], Joseph B. Ciolino[1*]

[1] *Department of Ophthalmology, Schepens Eye Research Institute of Mass Eye and Ear, Harvard Medical School, Boston, MA, USA*

[2]*Department of Ophthalmology, Massachusetts Eye and Ear; Infectious Disease Institute, Harvard Medical School, Boston, MA, USA*

[3]*The Charles T. Campbell Ophthalmic Microbiology Laboratory, UPMC Eye Center, Department of Ophthalmology, University of Pittsburgh School of Medicine, Pittsburgh, PA, USA*

[4]*Laboratory for Biomaterials and Drug Delivery, Dept. of Anesthesiology, Boston Children's Hospital, Harvard Medical School, Boston, MA, USA*

***Corresponding Author:**

Joseph B. Ciolino, Massachusetts Eye and Ear, 243 Charles St, Boston MA 02114

Tel: 617-573-5575 Email: joseph_ciolino@meei.harvard.edu

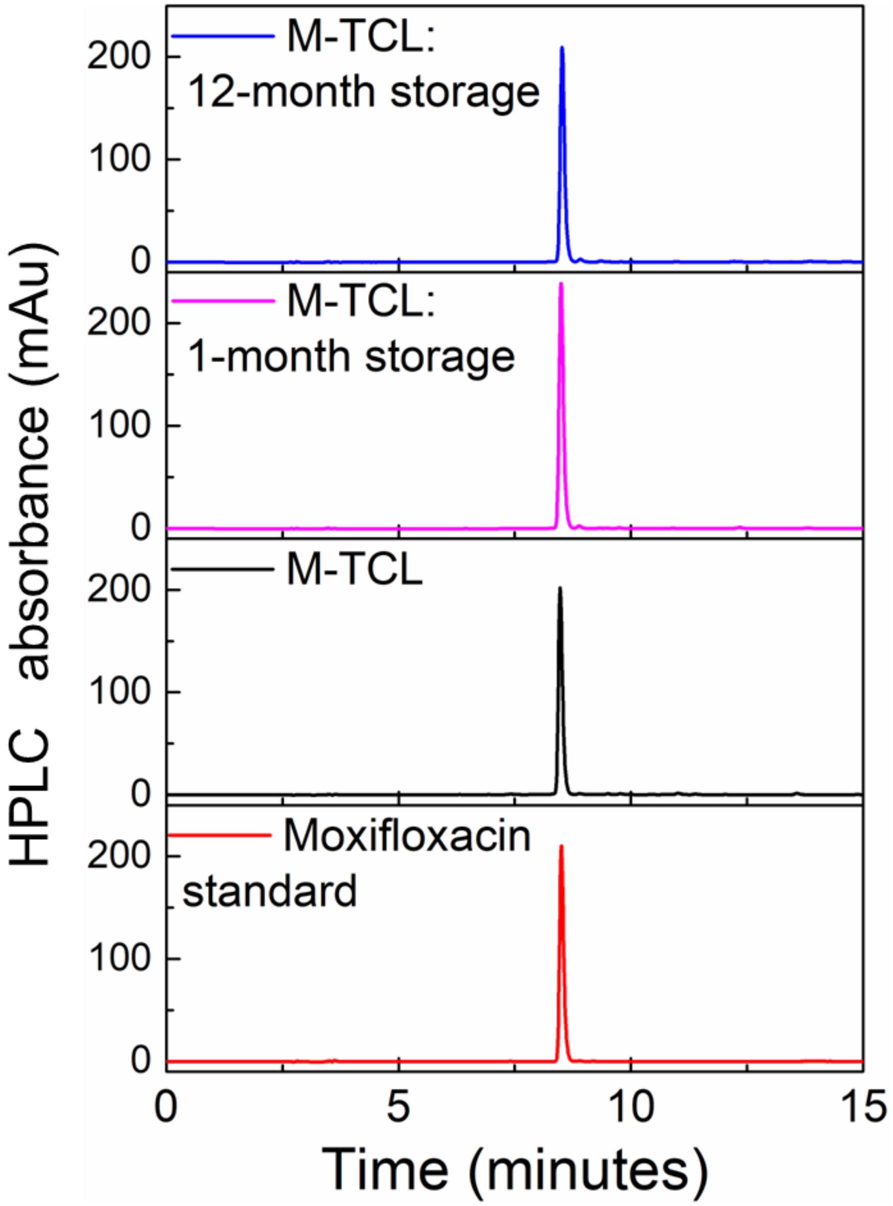

Figure S1. High-performance liquid chromatography (HPLC) chromatograms of moxifloxacin released from moxifloxacin-eluting therapeutic contact lenses (M-TCLs) after hydration, and after 1-month and 12-month storage in the storage solution at room temperature, were similar to that of neat pharmaceutical-grade moxifloxacin (standard).
